# Importance and variability of the paternal component in sow reproductive traits

**DOI:** 10.1007/s13353-024-00910-y

**Published:** 2024-10-18

**Authors:** G. Cieleń, E. Sell-Kubiak

**Affiliations:** Department of Genetics and Animal Breeding, Poznań University of Life Sciences, Wołyńska 33, 60-637 Poznań, Poland

**Keywords:** Additive genetic sire effect, Boar, Male reproduction, Non-genetic service sire effect, Swine

## Abstract

Reproductive traits are an integral part of the goals of the breeding programs that contribute to the economic success of production. Reproductive phenotypes such as litter size, number of piglets born alive, or litter weight at birth are mainly attributed to females. Thus, the maternal components can be found by default in quantitative genetics’ animal models. Still, paternal contribution to variance components should not be discarded. In this review, we indicate the importance of paternal effects in pig breeding by describing both the biology and genetics of boars’ traits, the use of (non-)genetic service sire effects in quantitative genetic models for traits measured on females, and genes involved in male reproduction. We start by describing the important biological traits of boars that have the most important effect on their reproductive abilities, i.e., sexual maturity, sperm quality, and testes parameters. Then we move to the possible environmental effects that could affect those traits of boars (e.g., feed, temperature). The main part of the review in detail describes the genetics of boars’ reproductive traits (i.e., heritability) and their direct effect on reproductive traits of females (i.e., genetic correlations). We then move to the use of both genetic and non-genetic service sire effects in quantitative models estimated as their percentage in the total variance of traits, which vary depending on the breed from 1 to 4.5% or from 1 to 2%, respectively. Finally, we focus on the description of candidate genes and confirmed mutations affecting male reproduction success: *IGF2*, *Tgm8*, *ESR1*, *ZSWIM7*, and *ELMO1.* In conclusion, the observed variance of paternal effects in female reproduction traits might come from various attributes of boars including biological and genetic aspects. Those attributes of boars should not be neglected as they contribute to the success of female reproductive traits.

## Introduction

Reproduction is the key to economic success in animal husbandry. In pig production properly, managed reproductive traits lead to a high number of healthy piglets in the litter. The litter size in pigs reached an average of 20 live piglets (SEGES–Danish Pig Science Centre 2020), and it is reported to still grow. Pig breeders are mostly focused on sows and their reproductive results, i.e., increasing the number of piglets weaned per year per sow and shortening the intervals between farrowings (Nowak et al. 2020). Many studies have concentrated on the total number born (Li et al. 2020; Ogawa et al. 2019; Ye et al. 2018) and piglet birth weight (Alves et al. 2018; Škorput et al. 2018; Moreira et al. 2020), defining these traits as the most informative phenotypic information on sow reproduction. In biological terms, the “female fertility” covers not only the ability of a sow to conceive but also to support the litter during prenatal and postnatal stages till weaning. This is reflected in phenotypic and genetic variation of economically important traits in pig breeding, e.g., litter size, and gives “female component” a wider range of expected effects. In the case of the “male fertility” or “male component,” it is usually limited to the sperm quality, testis size, and libido. However, when considering the genetic variation of reproductive traits in pigs, both maternal and paternal components can be found (Rothschild and Ruvinsky 2011).

In quantitative genetics, the animal model is used to estimate the variance components of the trait of interest and use it to obtain its heritability (Lynch and Walsh 1998.). Such models for reproductive traits in pigs (e.g., litter size and birth weight) always account for sow (maternal) additive genetic and maternal environmental variance (Bell et al. 2015). There is evidence that it is often a simplification, and the additive genetic and environmental variance also comes from the boar (paternal) side. This was observed by Zak et al. (2017) and Dobrzański et al. (2020) for litter size and by Sell-Kubiak et al. (2015) and Maiorano et al. (2019) for piglet birthweight. Moreover, in a meta-analysis of several genome-wide association studies, Sell-Kubiak et al. (2022a) indicated a link between a newly detected SNP for litter size and a gene affecting male reproductive performance in male rats. Thus, a better understanding of this paternal variance in the reproductive performance of pigs could allow more effective genetic improvement of those traits (Sell-Kubiak et al. 2022b).

Accordingly, we aim to perform a comprehensive review of possible sources of genetic and environmental paternal components in the reproductive traits of pigs by evaluating the biological impact of male reproduction on fertilization success, environmental impact on male reproductive traits, and ending with genetic aspects of male reproduction.

## Biological impact of male reproduction on fertilization success

Although the main focus of this review is on the genetic aspects of boars, it is necessary to start from the biological background of males of attributes of sexual maturity, sperm quality, and, ultimately, selection of the best boars for breeding programs.

### Maturity of the boar

In pig reproduction, despite many crucial genetic and environmental factors of the dam, the ability of the boar’s sperm to fertilize the eggs is also important (Banaszewska and Kondracki 2012). Table 1 shows the sperm characteristics affecting male reproductive performance. For most pig breeds, young males reach sexual maturity at the age of 6 months, but boars are used in the inseminator sector for at least 11 months (Stančić et al. 2012). The use of young animals for insemination is also not recommended due to low fertilization efficiency. In sexually mature boars, within the scrotal sac, the left testicle of a healthy boar hangs down lower than the right testicle. The weight of the testes is not affected by the future weight of the already mature boar. From the age of 9 to 10 months, ~ 145 kg of body weight boar testicles weigh between 310 and 360 g (Pinart and Puigmulé, 2013).

**Table 1:** Main male reproductive traits of interest for animal breeding (adapted from Rothschild and Bidanel, 1998)

Reproductive function	Main components of predictive traits
Sexual maturity	Age at first mating or sperm collection Testes size
Sexual behavior	Ability to mount
Gonadocyte production	Sperm quantity -Semen volume (per ejaculate, per day) -Sperm concentration -Testes size (length, width, circumference, volume and weight) Sperm quality -Sperm motility -Proportion of: *dead spermatozoa *abnormal spermatozoa
Fertility	Conception rate of mates
Prolificacy	Litter size of mates
Hormone levels	Testosterone level

Pubertal spermatogenesis, which refers to sperm formation through mature breeding life, is controlled by the hormones FSH, LH, and prolactin (Colin T. Whittemore 1998). LH is a pituitary hormone that stimulates the production of testicular steroids (and androgens), which are needed as part of the hormone complex supporting spermatogenesis (Rubin and Seebacher 2022). Once this process starts, the male exhibits clear sexual behavior. At the age of 6 months, a boar is still not fully developed in terms of other components of sexual libido that are important for stimulating gilts’ cyclical ovarian activity and oestrus behavior. This is because one of the important factors for effective stimulation of ovulation in gilts is the presence of a sufficient number of pheromones in boar saliva. These compounds are synthesized in submaxillary salivary glands (Stančić et al. 2012). The sexual development of young animals is important for improving ejaculation. In artificial insemination (AI) stations, attention is given to the libido of boars, which allows for frequent semen collection while minimizing complications (Lopez Rodriguez et al. 2017).

It is known that boars with higher libido produce ejaculates with more favorable parameter values, which means that appropriate stimulation can influence semen quality (Kondracki et al. 2021). Ultimately, a correlation exists between a boar’s level of libido expression and the characteristics of its ejaculate. Boars that take the longest time to initiate ejaculation tend to yield ejaculates with higher sperm concentration and quantity. The characteristics of ejaculate change with the duration of the semen collection interval (Savić and Petrović, 2015). The sexual activity of young males could serve as an indicator of their suitability for boar selection. The substantial genetic variance and heritability in the ejaculation rate and boar libido underscores the necessity of incorporating these traits into breeding programs. However, it also requires comprehensive knowledge of possible genetic and phenotypic correlations with other traits of interest to maintain the selection in the expected direction without negative trade-offs. The improvement in ejaculation performance as boars age is attributed to the maturation of testicular parenchyma and accessory sex glands (Kondracki et al. 2021).

### Sperm quality

In multiplications farms, pig breeders are paying more attention to the high quality of semen produced since most sows are artificially inseminated. Table 2 shows breed comparisons based on sperm concentration and seminal volume. There is substantial variation between the breeds in terms of the quality and quantity of semen. These differences are shown in the semen volume, semen concentration, and sperm motility. Most of the authors believe that most of these differences depend on the season and environmental factors such as temperature, intensity of lighting, moisture, and insolation (Waberski et al. 2019).

**Table 2:** Breed comparison based on selected semen traits

Specification	Duroc × Pietrain	Duroc	Pietrain	Hampshire
References	Knecht et al. 2014	Wysokińska and Kondracki 2013	Knecht et al. 2017	Wysokińska and Kondracki 2013
Seminal volume (mL)	245.1 ± 3.43	145.87 ± 44.78	236	229.24 ± 80.80
Sperm concentration (× 10^6^/mL)	391.8 ± 3.98	-	378.19	-
Total number of sperm (× 10^9^)	91.9 ± 1.04	83.80 ± 33.22	86.57	77.66 ± 27.02
Total number of live sperm (× 10^9^)	76.4 ± 0.89	76.32 ± 5.07	-	71.36 ± 4.10
Number of insemination doses (*n*)	24.47 ± 0.25	22.69 ± 7.74	23.12	23.02 ± 6.97

The semen quality is routinely analyzed with computer-assisted semen analysis (CASA). It allows precise evaluation of, e.g., motility and morphology of sperm, which leads to the proper selection of boars for artificial insemination (Dyck et al. 2011). Ejaculate testing guarantees good-quality semen, the use of which contributes to increasing the impact of valuable boars on genetic progress (Broekhuijse et al. 2015). The sperm mortality analysis is based on kinematic patterns, which have been linked to the reproductive traits of pigs (Barquero et al. 2021a). The number of sperm available for oocyte fertilization is essential for semen boar traits (Gomez Montoto et al. 2011). During the fertilization process, the major effect is sperm motility specifically the progressive motility of the sperm. Using an additional assessment of the selection of boars based on testicle size can improve boars’ reproductive traits and decrease the amount of straw needed for the insemination of sows (Jacyno et al. 2015).

Interestingly, testis size appears to be a good indicator of boar reproductive traits, such as sperm concentration, which holds significance for pork producers, especially considering the widespread use of artificial insemination on most farms. Jacyno et al. (2015) reported notably high phenotypic correlation coefficients between the width of the left testis and the sperm concentration, total sperm count, and percentage of progressively motile sperm. Additionally, the width of the left testis exhibited strong phenotypic correlations with the volume of both the left and right testis, as well as the total testicular volume. Although a positive phenotypic correlation was found between the left and right testicles, it should be noted that the highest phenotypic correlation coefficients were found between the width of the left testis. Moreover, in the same study by Jacyno et al. (2015), it was confirmed that there is no negative genetic correlation between selection for increases in test size and carcass traits. These results suggest that straightforward measurements of testicular dimensions, particularly the width of the left testis, can serve as reliable indicators for estimating the reproductive performance of boars.

### Selection of the best boar and genetic improvement with AI

In a fully functional breeding program or on a commercial farm, the selection of a boar is an extensive process that requires the collection of biological and genetic information on the boar. This involves a very detailed leg and confirmation check, semen quality evaluation, and reproductive health check. In addition, the breeding values are estimated based on their own performance (e.g., growth rate) and female sibling and half-sib performance for reproductive traits. Taken together, these findings present the potential of the boar to contribute to genetic gain in the next generation.

The implementation of artificial insemination (AI) with fresh semen has had a large impact on the selection intensity in swine breeding programs. This has significantly reduced the number of boars in the herd while at the same time increasing the fertility and genetic value of each boar (Oh et al. 2005). To select excellent boars for AI, the best linear unbiased prediction (BLUP or GBLUP, when genomic information is available) was used.

### Environmental impact on male reproductive traits

The quality of semen is affected not only by the boar genotype (which will be evaluated in the next section) but also by many environmental factors, such as season, feeding, health issues, management, housing, and the Al station (Schulze et al. 2014). During the processing of semen collection, boars may be prone to stress; therefore, it is essential to develop feeding strategies to reduce mortality (Lopez Rodriguez et al. 2017). Another main unfavorable factor is heat stress during the summer, as well as seasonality, which causes a decrease in sperm quality. The reduced production outputs observed during heat stress could be attributed to various underlying mechanisms, such as reduced feed intake, altered metabolic rate, and flexibility (Serviento et al. 2020). Specifically, in comparison to animals on the same nutritional regime, heat-stressed pigs exhibit greater adipose tissue deposition at the price of lean tissue accretion (Rudolph et al. 2024).

Heat stress can have rapid consequences. Semen quality and quantity can be significantly reduced by exposure to ambient temperatures above 30 °C for a short duration, such as 72 h. After acute heat stress, normal sperm production usually declines within 2 weeks and takes an extra 4–6 weeks to reach prestress levels (Flowers 2022). It is worth ensuring good housing conditions for boars with bedding material but also providing cooling systems during heat treatment (Cabezón et al. 2016). The next important aspect is hygienic sperm collection. Not following these rules, the semen dose can result in bacterial contamination. This influences not only the quality but also antimicrobial resistance in isolates from extended semen. It is necessary to monitor bacterial and viral pathogens not only due to decreased semen production but also to avoid the risk of herd infection (Kuster and Althouse 2016). Boar semen obtained under normal circumstances has a variety of aerobic microorganisms, including fungi (37.5%) and bacteria (62.5%). The bacterial contamination of AL can be considerably decreased by the use of antibiotics in commercial extenders and strict hygiene practices during semen collection and processing (Ciornei et al. 2021). In recent years, numerous studies on the management of swine semen at low temperatures have been published. This demonstrates that antibiotic-free low-temperature storage is feasible, inhibits bacterial development, and yields fertility results comparable to those of traditional storage at 17 °C (Hensel et al. 2023). The process by which collecting sperm is exposed to many stress factors can cause oxidative stress and capacitation changes, decreasing fertility potential. Additionally, the dilution rate or dilution temperature can affect the quality of semen. High-quality packing may avoid plastic, which can be toxic to semen. By following appropriate procedures et al. stations, the causes described above could be avoided, ensuring high-quality semen production (Malama et al. 2017).

The effect of boar semen characteristics on reproductive traits, e.g., the farrowing rate and the total number and number of stillborn pigs at birth, was measured by Park (2013). Her study showed that not only sows but also boars have an impact on reproductive traits. A study by Flowers (2015) divided efficient sperm production into two components: developmental and functional. Developmental components have a permanent effect on the production of sperm. The birthweight was considered the best selection tool because it has a positive genetic correlation with adult sperm production. This means that boars of appropriate birthweight produce a greater amount of semen after reaching sexual maturity, influencing fertilization (Francois et al. 1997). The functional components, such as collection frequency, housing, nutrition, and photoperiod, which can have genetic and negative effects on sperm production, have temporary effects on spermatogenesis. The authors emphasize that enhancements in one of these areas may be capable of offsetting deficiencies in others. The feeding strategy should be set up so that young boars (until they are 7 months old) can only obtain 400–600 g each day. This involves adding vitamins, minerals in both organic and inorganic forms, fatty acids, and amino acids. These micronutrients are usually either components of the sperm or crucial components or cofactors in vital metabolic pathways required for the protection, growth, and function of sperm. Thus, it seems logical that adding micronutrient supplements to a balanced diet could improve spermatogenesis (Kleve-Feld et al. 2024). When other conditions are introduced that are expected to have a negative impact on sperm production, including heat stress, frequent semen collection, or other kinds of stressors, micronutrient supplements increase semen quantity and quality. Semen quality was found to improve with the addition of vitamins A and E, selenium, and arginine (Flowers 2022).

## Genetic aspects of male reproduction

The ability of males to provide large and healthy litters has not been studied as broadly as it has been for sows since reproductive pressure on all livestock species is placed on females. Additionally, as mentioned in the previous section, male reproductive traits include sperm quality and libido rather than the number of piglets. In contrast, sows’ reproductive traits include the farrowing rate (Kohn et al. 2018; Nabi et al. 2004), the number of piglets born alive (Popwell and Flowers 2004; Gadea 2003), litter weight at birth (Hassanien and Baiomy 2011; Ogawa et al. 2022), and stillborn or mummified piglets (Schoos et al. 2023; Wang et al. 2021). Even though the improvement of female reproductive traits was mainly possible by a high selection intensity of males, females and the traits collected directly on them play such important roles in pig breeding programs.

### Heritability of male reproductive traits and their effect on female reproduction

Genetic improvement of both female and male reproductive traits in pigs is challenging due to their low heritability (*h*^*2*^ ~ 0.01–0.20; Sanglard et al. 2019). The estimates of h^2^ for the male reproductive traits are presented in Table 3 (note: here we focus on a classical interpretation of the “male component/fertility” being linked with boar’s own phenotype). Generally, the estimates of heritability (h2) and repeatability (r2) for reproductive traits directly measured on boars are low to moderate, consistent with findings in the literature for other species. This suggests there is less genetic variance hence potential effects of selection are limited compared to environmental factors which have a stronger influence on reproductive traits. The same is true for sows and their main reproductive traits of interest. Thus, the identification of an alternative trait that is correlated with the reproductive performance of sows and is highly heritable could be used as a tool to improve reproductive efficiency in pigs (Zak et al. 2017). One of the options for such a trait could be a male fertility indicator that is both easy to measure and has a relatively high h^2^. It would be interesting to identify sires to improve the reproductive success of sows. However, in reality, more than one indicator would be needed, and preferably those would be included in the breeding value of the boar to provide a broad overview of his abilities.

**Table 3:** Heritability estimates for male reproductive traits in different pig breeds

Trait	Heritability (h^2^)	Genotype	Reference
Testicular size: Testis width Testis area	0.66 0.69	Landrace Yorkshire	Sanglard et al. 2019
Semen volume	0.25 0.20	Landrace Large White	Wolf 2010
Semen motility	0.07–0.17	Duroc	Chang et al. 2017
Sperm concentration	0.20	Duroc	Ogawa et al. 2022
Libido	0.12–0.20	Duroc Landrace Yorkshire	Chang et al. 2017
Sex hormones (testosterone and estradiol)	0.17 to 0.29	Pietrain × Large White Pietrain	Parois et al. 2015
Abnormal sperm percentage	0.05–0.24 0.06–0.34	Landrace Large White	Hong et al. 2022

A good example of such a male fertility indicator could be testis size, which has high heritability (0.20–0.70). This trait is also straightforward to measure and can be assessed at a young age (Sanglard et al. 2019). Leveraging testes size as a selection criterion could yield indirect improvements in reproductive traits in sows, particularly if these traits exhibit substantial genetic correlations. Further research is needed to explore potential genetic correlations thoroughly. Additionally, male traits such as testis or accessory gland measurements might respond more easily to selection than sperm volume, libido traits, and testosterone levels, which are less heritable (Rothschild and Bidanel 1998). Sanglard et al. (2019) reported a linear phenotypic relationship between reproductive traits in sows and the testis area and width in boars.

Larger testes are associated with higher levels of various hormones throughout male development, such as luteinizing hormone (LH) (Sanglard et al. 2019). The authors showed that the reduction in several survival traits was linked to a large TA. At the same time, Sanglard et al. (2019) showed that a testis area in a range of 15.8–16.3 cm^2^ increases the survival of piglets while minimizing their mortality. As a result, an increase in the total number of individuals born is observed from 17.2 to 18.5. In relation to TW, also the survival traits increased linearly (*P* < 0.05) as TW rose; however, piglets mortality did not (*P* ≥ 0.11) (Sanglard et al. 2019). Phenotypic assessment of the testis width of sires is a useful tool for selecting animals for breeding and improving female performance (Sanglard et al. 2019). Additionally, these findings raise the possibility of indirect early selection for female traits in boars. However, to apply this approach, the study of Sanglard et al. (2019) should be repeated on a larger population to obtain accurate positive genetic correlations to avoid undesirable consequences of such selection.

A positive correlation was also shown between maternal traits and several kinematic parameters of boar semen, such as curvilinear velocity (Hong et al. 2018; Winters et al. 2018), straight-line velocity (Kwon et al. 2015), and beat cross frequency, according to kinematic variables (Barquero et al. 2021b). Similar correlations between semen traits and female reproductive traits were also observed in rabbits (Quintero‐Moreno et al. 2007; Nishijima et al. 2015; Theau-Clement et al. 2016). In pig production, the effects on fertility traits have been observed in terms of kinematic patterns and morphological variables (Henning et al. 2022; Tremoen et al. 2018). The morphometric characteristics of the ejaculate include a heterogonous population of spermatozoa with different effects on fertilization. Some authors have studied spermatozoa morphology and directly correlated sperm head size with sow fertility traits, e.g., the weaning-to-first-mating interval and prolificacy (Barquero et al. 2021b).

Examples of the genetic correlations between testis size and female reproductive traits are presented in Table 4. Depending on the publication and the male/female traits of interest, those genetic correlations vary from − 0.034 for sperm concentration and a number of stillborn piglets (Ogawa et al. 2022) to 0.39 for testes size and total number born (Ytournel et al. 2014).

**Table 4:** Genetic correlation between semen traits and female reproductive traits

Traits	Semen volume	Sperm concentration	Proportion of morphologically normal sperm	Testes size
	Ogawa et al. (2022)			Ytournel et al. (2014)
Total number born	0.09	− 0.26	0.12	0.39
Number born alive	0.12	− 0.20	0.18	-
Number stillborn	− 0.10	− 0.34	− 0.17	-
Litter size at weaning	0.25	− 0.08	0.26	-
Survival rate at birth	0.19	0.31	0.22	-
%piglets born alive	-	-	-	0.30
%piglets weaned	-	-	-	0.10

### Paternal effects on phenotypic variation in sow reproductive traits

In quantitative genetic models (i.e., statistical animal models) for reproductive traits (piglets’ birthweight or litter size), maternal variation is included by default (Lough et al. 2017). This variation is strongly linked to the characteristics of different maternal lines (Mazzatenta et al. 2021); thus, different levels of heritability are present for those traits depending on the breed of the sow. At the same time, the effects of male sex on reproductive traits are often not considered at all or are even disregarded (Dobrzański et al. 2020; Zak et al. 2017). This took place even though a substantial number of studies reported the paternal effect as significant (“service sire effect”; Table 5) or having a genetic effect on top of the environmental effect (“additive genetic sire effect” or “genetic service sire”; Table 5). In both cases (genetic and environmental), the effect of the paternal trait varied from 1–4.5% of the total phenotypic variance of the trait. Notably, in most cases, the addition of those effects improved the quality of the model for the reproductive traits of sows (e.g., Sell-Kubiak et al 2015 and Cieleń et al. 2023). In the study of Sell-Kubiak et al. (2015), there were not only noticeable paternal effects on birthweight but also considerable estimates of sire variance for the variability of birthweight. Thus, it is clear that reproductive traits that are mostly attributed to the mother can also be affected by the paternal side. In most cases, however, paternal effects were disregarded because they explained a relatively low proportion of the total variation in the trait, not greater than ~ 3% (Park 2013).

**Table 5:** Percentage of phenotypic variance of reproductive traits explained by (service) sire environmental and genetic variance

Traits	Sire additive genetic variance	Service sire effect	Breed	Source
Number born alive	-	4.5%	Złotnicka White	Sell-Kubiak et al. (not published)
	-	5.5%	Złotnicka Spotted	Sell-Kubiak et al. (not published)
	-	1%	Finnish Landrace	Serenius et al. 2004
	-	2%	Large White	Serenius et al. 2004
Total number born	-	1%	Finnish Landrace	Serenius et al. 2004
	-	2%	Large White	Serenius et al. 2004
	1%	3%	Landrace	Cieleń et al. 2023
	-	2%	Czech Landrace	Wolf and Wolfova 2012
	-	2 – 3%	Czech Large White	Wolf and Wolfova 2012
Number of piglets weaned	-	2%	Finnish Landrace	Serenius et al. 2004
	-	2%	Large White	Serenius et al. 2004
Birth weight	2%	-	Landrace x Large White	Sell-Kubiak et al. 2015
	2%	-	Large White x Landrace	Sell-Kubiak et al. 2015

In terms of statistical modeling, it has also been shown that the paternal effect should not be ignored in animal models to obtain valid results for estimating variance components. The main reason for this difference is the repeated records used per sire; thus, to properly account for sire genetic variance, permanent service sires should also be included (Van der Lende et al. 1999; Serenius et al. 2003; Wolf and Wolfova 2012). However, there are also other statistical reasons for this. Varona et al. (2015), who based their research on beef cattle data, described some controversy that may occur when animal models include only maternal effects, which results in strongly negative estimates of covariance between direct and maternal effects. Meyer and Tier (2012) also indicated in their research on cattle that ignoring paternal effects leads to incorrect results in the estimates of variance components (5–7% of the phenotypic variation for birth and weaning weights). Moreover, the use of a model based on service sires could lead to the estimation of sire-based repeatability in sow reproductive traits. This was done in pigs, e.g., by Van der Lende et al. (1999) and Serenius et al. (2003); however, it is much more broadly studied in cattle (e.g., Varona et al. 2015; Meyer and Tier 2012). The repeatability obtained for pig reproductive traits is low, as presented in Table 3; thus, it would be very difficult to include them in the selection process.

In addition, the quantitative genetic models allow the correlation between the model terms for a certain trait to be incorporated. Several authors, such as Van der Lender et al. (1999), Serenius et al. (2003), and Wolf and Wolfova (2012), have evaluated the relationships between service sire effects and other variances in the model. Their findings indicate that the correlation between the additive genetic effect of the dam and the additive genetic effect of the service sire for the total number born is negative and varies from − 0.11 to − 0.47 depending on the breed. Thus, ignoring the effect of the boar on the reproductive traits of the sow is not optimal because it is antagonistic to the service sire effect.

### Known genes associated with male (and female) pig reproduction

Many studies have revealed genes with clear effects on male reproduction. One of the genes of interest in male reproduction is *IGF2*, which plays a role as a prenatal growth factor. Aad et al. (2013) described the ability of *IGF2* to stimulate steroidogenesis and mitogenesis within bovine follicles. Nordin et al. (2014) showed that *IGF2* is uniquely activated by paternal genes in the mouse placenta and is crucial for the normal growth of pups during prenatal development. Dean (2013) showed that the *Tgm4* gene affects the viscosity of semen and has a major effect on male reproductive success. In Duroc pigs, Zhang et al. (2023a, b, c) found significant candidate genes related to sperm motility, namely, *STRA8*, *ZSWIM7*, *TEKT3*, *UBB*, and *CATSPER.* The *TEKT3* is required in mice for proper sperm motility, whereas the mutation in *the UBB* gene results in male and female infertility in mice. The *CATSPER1* protein is expressed during meiosis and postmeiosis in human testicular tissue and is localized in the sperm tail.

An interesting candidate gene for improving male fertility traits is *ESR1* (also known for its effect on sow reproduction). Its role in the function of this gene was initially described by Gunawan et al. (2011). The authors described reproductive problems in males due to the lack of *ESR1*. Evidence for this phenomenon includes *ESR1* knockout mice, which exhibit deficiencies in maintaining proper spermatogenesis. In the future, the use of this gene in the breeding boar selection process could be supported by the correlation between *ESR1* polymorphisms and the fertility and quality of boar sperm. In the Landrace breed, *COX-2* gene important for male fertility is at the same time associated with the total number born (Zhang et al. 2023a, b, c). Those authors also indicated the *ELMO1* gene which is involved in both spermatogenesis and the clearing of apoptotic germ cells in female mice. Additionally, in the Duroc breed, three candidate genes for male reproductive performance, *PLC*_*z*_, *VWF*, and *ZP3*, were shown to be significantly associated with the total number of piglets born (Tremoen et al. 2018), whereas Tremoen et al. (2019) showed that in Landrace *BMPR1B* gene is highly relevant for male and female fertility traits.

Ultimately, these findings contribute to describing the genetic foundation of porcine reproductive characteristics and suggest molecular regions as indicators for genomic selection in male pig breeding.

#### Genome-wide association studies

Moreover, genome-wide association studies (GWAS) have identified several QTLs and candidate genes related to boar semen traits (Zhao et al. 2020) but also in other livestock species, such as sheep (Serrano et al. 2021; Hodge et al. 2023), goats (Wang et al. 2020; Talouarn et al. 2020), and cattle (Fonseca et al. 2018; Qin et al. 2017). Table 6 shows the most significant candidate genes for different pig breeds. Candidate genes have been reported for both sperm quantity, i.e., the number of sperm (Gao et al. 2019; Kaewmala et al. 2011; Brinke et al. 2020), and quality, i.e., morphology abnormalities and sperm motility (Zhao et al. 2020; Marques et al. 2018; Diniz et al. 2014). A GWAS of Duroc boar revealed that single nucleotide polymorphisms (SNPs) on chromosomes 9, 12, 11, and 16 are associated with the number of sperm cells, sperm motility, morphological abnormalities, and progressive motility (Zhao et al. 2020). In the same study, the *NIMA* gene was identified as a possible candidate gene for these traits based on previous reports demonstrating its role in the process of the meiotic cell cycle and the first meiotic division in mouse spermatocytes. Another interesting candidate gene was found by Sell-Kubiak et al. (2022a, b), as indicated by pCADD post-GWAS analysis of litter size in pigs. The promising candidate gene indicated by those authors was *SOSTDC1*, which has a proven effect on male fertility in rats. Sell-Kubiak et al. (2022a, b) emphasized that the effect of this gene has also been proven in rats. This is a rather surprising discovery since, as mentioned earlier, litter size is linked with the female sex and less closely to the male sex.

**Table 6:** Candidate genes associated with semen traits in different pig breeds

Trait name	SSC^a^	Gene	Function	Breed	Reference
Sperm motility	1	*ESR1*	Sperm morphology and motility	Pietrain, Hampshire	Cooke et al. 2017
	1	*ESR2*	Spermatogenesis	Pietrain	Gunawan et al. 2012
	5	*CD9*	Sperm–egg fusion	Pietrain, Hampshire	Kaewmala et al. 2011
	5	*SCN8A*	Progressive motility	Large White	Gmel et al. 2021
	7	*SPATA7*	Spermatogenesis	Landrace	Kannabiran 2020
	12	*DNAI2*	Sperm motility	Large White	Hu et al. 2023
	13	*IQCG*	Sperm flagellum formation	Large White	Li et al. 2014
Semen volume	1	*ESR2*	Spermatogenesis	Pietrain	Gunwan et al. 2012
	7	*C7H15orf39*	Semen quality traits	Duroc	Wang et al. 2023
	9	*PIWIL4*	Prospermatogonial development	Landrace	Song et al. 2015
Sperm progressive motility	5	*SCN8A*	Progressive motility	Large White	Gmel et al. 2021
	7	*METTL3*	Male fertility and spermatogenesis	Landrace	Xu et al. 2017
	8	*HPGDS*	Regulation of the spermatogenesis	Landrace	Jastrzebski et al. 2022
	13	*IQCG*	Sperm Flagellum Formation	Large White	Li et al. 2014
Sperm per ejaculate	2	*NME5*	Mitosis and meiosis	Landrace	Liu et al. 2020
	7	*TEP1*	Male infertility risk	Duroc, Erhualian	Yan et al. 2014
Sperm abnormality rate	5	*CD9*	Sperm–egg fusion	Pietrain, Hampshire	Kaewmala et al. 2011
	6	*AZIN2*	Testosterone synthesis	Landrace	Lambertos et al. 2018
	7	*SPATA7*	Spermatogenesis	Landrace	Kannabiran 2020
	12	*NOS2*	Semen quality traits	Duroc	Wang et al. 2023
Sperm concentration	1	*ESR2*	Spermatogenesis	Pietrain, Hampshire	Gunawan et al. 2012
	5	*PLCZ*	Sperm and embryonic development	Pietrain, Hampshire	Eum et al. 2022
	7	*TEP1*	Male infertility risk	Duroc, Erhualian	Yan et al. 2014
	9	*PILWIL4*	Prospermatogonial development	Duroc	Song et al. 2015

^a^*SSC* Sus scrofa chromosome

#### Genes in other species

In other livestock species, several studies have been conducted to determine the genetic background of male reproductive traits, mostly concentrating on sperm quality. Considering candidate genes related to semen volume and the total number of sperm cells in Holstein–Friesian bulls, GWAS identified *PHF7*, *TLR9*, and *SPATA7*, which have been previously reported to be involved in spermatogenesis (Gao et al. 2019). In another association study by Sweett et al. (2020) involving beef cattle, 14 candidate genes related to male fertility were identified. The authors highlighted two genes associated with scrotal circumference, MAP3K1 and VIP, which are involved in regulating testicular function. The authors also suggested that these genes could be potential biomarkers for spermatogenesis and apoptosis. In the same study, several other candidate genes, i.e., *SOD2*, *TCP1*, *PACRG*, *SPEF2*, and *PRLR*, associated with sperm quality, such as concentration and development, were also identified. A study on rooters by Sun et al. (2019) revealed two candidate genes associated with sperm motility. First, the *H-PGDS* gene is important for prostaglandin-D synthase activity. In the second pathway, WNT2 is involved in the development of seminal vesicles. The authors also performed qPCR for these two genes. Due to impaired spermatogenesis in these birds, low expression of the *H-PGDS* and W*NT2* genes indicated inhibition of prostaglandin D2 synthesis, which explained the low sperm motility. A poultry study by Zhang et al. (2023a, b, c) revealed four candidate genes related to semen volume for roosters: *FAPP1*, *OSBPL6*, *SESTD1*, and *SSFA2*. The authors point to chromosome 7, which exhibited 197 significant SNPs, among which only four genes had known functions related to semen volume. The *FAPP1* gene is associated with *Golgi* membrane transport, where vesicles from the membrane form the sperm acrosome. The function of the *OSBPL6* gene has been linked to embryo quality, suggesting that this gene may contribute to spermatogenesis and, consequently, regulate semen quality. The next gene, *SESTD1*, is related to azoospermia, and the last gene, *SSFA2*, is confirmed to play a significant role in acrosome formation in sperm.

#### Paternally expressed genes

Another aspect is paternally expressed imprinted genes (PEGs, Davies et al. 2005). Genomic imprinting frequently leads to parent-of-origin-specific differential expression of alleles inherited from the mother and father, and it is crucial for mammalian development and growth. For instance, a distinctively methylated region (DMR) located at the *SGCE/PEG10* locus functions as the imprinting control region (ICR), ensuring paternal allele-specific expression across species, including humans (Jiang et al. 2015; Soubry et al. 2016) and mice (Soubry et al. 2015). Despite extensive studies in mice and humans, our understanding of genomic imprinting in pigs remains limited (Wu et al. 2020).

#### Heterosis effects and epigenetic on male reproductive traits

Boars’ sperm quality is influenced by both extrinsic (environmental/husbandry) and intrinsic (genetic) effects. The significance of paternal heterosis in this context is demonstrated by the greater reproductive efficiency of crossbred boars than of purebred boars concerning intrinsic parameters (Pinart and Puigmulé, 2013). Even though heterosis is highly useful in pig breeding, there are very few studies evaluating the impact of crossbreeding on boar reproductive qualities, and the majority of these studies rely on relatively small datasets or a limited sample size of males (Smital et al. 2004). Substantial differences in the seminal quality of boars have been observed in numerous studies involving different pure genetic lines, while other studies have shown that crossbred boars often generate the best seminal quality, followed by purebred maternal lines and purebred terminal lines (Pinart and Puigmulé, 2013). The susceptibility to seasonal infertility, ejaculate volume per age interaction, optimal collection frequency, rate of ejaculates discarded owing to poor quality, age of puberty, libido, trainability, and sperm lifetime are further different between purebreds and crossbreds (Vidocić et al. 2012). Table 7 shows the differences in heterosis effects on semen characteristics.

**Table 7:** Heterotic effects of different crosses of pig breeds on semen characteristics (Pinart and Puigmulé, 2013)

Cross	Semen traits	Heterotic effect
Duroc × Large white	Semen volume	3%
	Sperm concentration	4%
Large white × Pietrain	Semen volume	4% or 6–7%
	Total number of spermatozoa/ejaculate	3%
Duroc × Hampshire	Semen volume	23% or 12%
	Total number of spermatozoa/ejaculate	8%
	Sperm motility	2%
Hampshire × Pietrain	Semen volume	32% or 12%
	Total number of spermatozoa/ejaculate	18% or 6%
	Sperm viability	10%
	Sperm motility	2%
Duroc × Pietrain	Semen volume	6–7%

Changes in gene function that are heritable without affecting DNA sequence are known as epigenetics (Vodička et al. 2005). Epigenetics is crucial for comprehending the diversity and inheritance of complex traits in pigs. DNA methylation has been investigated as a significant epigenetic alteration linked to pigs’ growth (Hu et al. 2018), immunological response (Wang et al. 2017), and reproduction traits (Congras et al. 2014). Investigating the epigenetics of the testis in pigs may enhance our comprehension of male fertility and sexual maturity in boars. The epigenome of the testis is crucial for investigating the hereditary transmission of boar taint in pigs, an undesirable odor that arises from cooking pork from uncastrated male pigs and is genetically inherited (Wang and Kadarmideen 2019).

## Conclusion

This review reveals the importance of paternal effects in the reproductive traits of pigs starting from the biological aspects, through environmental factors affecting boars’ fertility, and finishing with the genetic background described in both quantitative and molecular terms. Proper management of a boar from the moment it reaches sexual maturity is a crucial factor in its ability to function as a successful breeder. Since most sows are artificially inseminated, pig breeders concentrate more on the better quality of produced semen. Improving ejaculation requires that the young animals complete their sexual development, as boars’ libido is important for regular semen collection in artificial insemination stations. The quality of semen is affected by many environmental factors, such as season, feeding, health issues, management, housing, and the Al station. Some studies show that testes size seems to be a reliable predictor of boar reproductive characteristics, such as sperm concentration, given the prevalence of artificial insemination on most farms. It is noteworthy that testes size is also linked with female reproductive traits, e.g., total number born, and could be used as an indicator of female reproduction based on easy-to-evaluate male characteristics with moderate heritability. Furthermore, the service sire effect does not have the same magnitude in all breeds and traits. The paternal effects from the animal model were estimated on the level of 1–2% for additive genetic effect and 1–4.5% for environmental effect. The explanation could be that indeed the paternal effects are more environmental based than genetic when it comes to female reproduction traits. Nonetheless, the paternal, i.e., service sire effects, should not be ignored in the quantitative genetic models as ignoring them can lead to incorrect estimates of the remaining variance components and thus affect the heritability and breeding value estimations. Moreover, several candidate genes were already indicated with potential direct effects of the boar on female reproduction traits: *IGF2*, *Tgm8*, *ESR1*, *ZSWIM7*, and *ELMO1*. Such knowledge of specific genomic variation can be beneficial in breeding programs to select the animals with certain variants that benefit both male and female fertility.

In conclusion, the observed variance of paternal effects in female reproduction traits might come from various attributes of boars including biological and genetic aspects. Those attributes of boars are worth further explorations as they contribute to the success of female reproductive traits and thus the entire pig breeding industry.
